# Development of Triazoles and Triazolium Salts Based on AZT and Their Anti-Viral Activity against HIV-1

**DOI:** 10.3390/molecules26216720

**Published:** 2021-11-06

**Authors:** Daniel Machado de Alencar, Juliana Gonçalves, Andreia Vieira, Sofia A. Cerqueira, Cruz Sebastião, Maria Inês P. S. Leitão, Giulia Francescato, Paola Antenori, Helena Soares, Ana Petronilho

**Affiliations:** 1 ITQB-NOVA—Instituto de Tecnologia Química e Biológica António Xavier, Universidade Nova de Lisboa, Avd da Republica, 2780-157 Oeiras, Portugal; dalencar2497@gmail.com (D.M.d.A.); andreia.sdc.vieira@gmail.com (A.V.); inesleitao@itqb.unl.pt (M.I.P.S.L.); giulia@itqb.unl.pt (G.F.); paola.antenori@gmail.com (P.A.); 2 Human Immunobiology and Pathogenesis Laboratory, Chronic Diseases Research Center, NOVA Medical School, NOVA University of Lisbon, 1150-082 Lisbon, Portugal; juliana.goncalves@nms.unl.pt (J.G.); sofia.cerqueira@nms.unl.pt (S.A.C.); cruzdossantos10@gmail.com (C.S.)

**Keywords:** click chemistry, triazoles, triazolium salts, anti-viral, HIV-1, AZT

## Abstract

We report herein a set of 3′-azido-3′-deoxythymidine (AZT) derivatives based on triazoles and triazolium salts for HIV-1 infection. The compounds were synthesized via click chemistry with Cu(I) and Ru(II) catalysts. Triazolium salts were synthesized by reaction with methyl iodide or methyl triflate in good yields. The antiviral activity of the compounds was tested using two methodologies: In method one the activity was measured on infected cells; in method two a pre-exposure prophylaxis experimental model was employed. For method one the activity of the compounds was moderate, and in general the triazolium salts showed a decreased activity in relation to their triazole precursors. With method two the antiviral activity was higher. All compounds were able to decrease the infection, with two compounds able to clear almost all the infection, while a lower antiviral activity was noted for the triazolium salts. These results suggest that these drugs could play an important role in the development of pre-exposure prophylaxis therapies.

## 1. Introduction

The extraordinary development of therapies for Human Immunodeficiency Virus (HIV) treatment over the last decades has extensively reduced the morbidity and mortality associated with the disease [1]. At present, there are over 30 drugs utilized for the treatment of HIV, divided into five main classes, which target different steps in the viral life cycle: (1) viral entry; (2) reverse transcription; (3) integration; (4) viral maturation [2]. Prophylactic treatments are also available, reducing considerably the risk of infection, either in pre-exposure and in post-exposure situations [3]. However, current therapies are compromised by the rapid emergence of resistant strains, side effects resulting from extended use of drugs, and their poor bioavailability [4,5,6]. The discovery that HIV-1 continues to replicate even in the patients under anti-retroviral therapy [7,8] has led to an urgent need to develop novel anti-HIV drugs with increased efficiency, minimum side effects, and higher bioavailability. Indeed, recent studies identified a specific T cell subset, the follicular cells (Tfh), as the major source of replication-competent HIV-1 in antiretroviral-treated patients [9,10]. 

3′-azido-3′-deoxythymidine (AZT, zidovudine) was the first approved drug for the treatment of the Human Immunodeficiency Virus (HIV) [4]. AZT is a nucleoside RT inhibitor, which lacks the 3′-OH group of the deoxyribose moiety and operates as an obligate chain terminator. AZT was initially used as a standalone drug and later as part of the highly active antiretroviral therapy (HAART) drug combination, with lamivudine and abacavir [11]. Long-term use of AZT is associated with a significant number of side effects, including myopathy, cardiomyopathy, and anemia, among others [5], which result from the ability of AZT to be both a substrate and inhibitor of human thymidine kinases. AZT can also be used to prevent HIV infection and has been used, for instance, to reduce mother to child transmission [12].

Appropriate modification of AZT can be used as a tool to increase the efficiency of the drug and introduce additional functionalities to further expand its range of activity. For example, modifications of AZT within the sugar can activate or block a specific function [13]. The presence of an azide group makes AZT a suitable substrate for further modification via Huisgen 1,3-dipolar cycloaddition to form triazoles, known to be active as antivirals in their own right [14]. Accordingly, the synthesis of AZT modified with 1,4- and 1,5-triazoles can be easily achieved using copper [15] or ruthenium catalysts [16]. This possibility has been explored previously. Earlier reports on the antiviral activity of AZT modified with 1,4 triazoles bearing alkyl groups indicated these compounds are not effective as antiviral agents for HIV-1 [17,18]. The combination of AZT with HIV inhibitors via click chemistry was also reported by Danel et al. [19]. Yet, the resulting dimeric compounds only showed moderate activity against HIV. More recently, Wang et al. reported the first series of AZT-derived 1,2,3-triazoles with low sub-micromolar activities against HIV-1 [20]. The active AZT derivatives present a 1,5- connectivity at the triazole ring, as well as a bulky substituent. Although less effective, 1,4-triazoles with sterically demanding substrates also showed anti-viral properties. 

Following our work on the modification of nucleosides [21,22,23], herein we report the synthesis of 1,4- and 1,5-AZT derivatives based on triazoles, and their corresponding triazolium salts, and examine their activity against HIV-1, utilizing two different methodologies. Specifically, we examine the different activities of 1,4- and 1,5-triazoles, as well as their corresponding triazolium salts, exploiting the possibility of using these compounds to prevent HIV infection. 

## 2. Results

### 2.1. Synthesis and Characterization

**Scheme 1:**
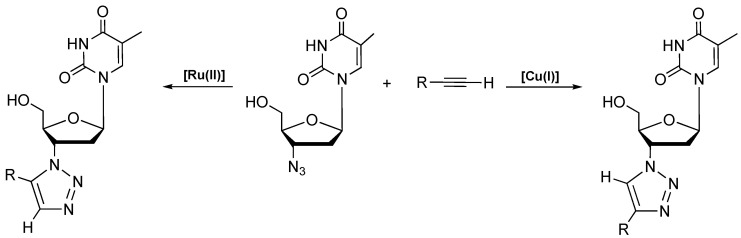
Modification of AZT via click chemistry with copper(I) and ruthenium(II) catalysts.

We synthesized 1,4-triazoles and 1,5-triazoles via 1,3-Huisgen cycloaddition with different substituents, using Cu(I) and Ru(II) catalysts, respectively [15,16]. For 1,4-triazoles, AZT reacts with the corresponding alkyne with the Cu(I) catalyst (5%) at 100 °C for 2 h, in *tert*-butanol. For 1,5-triazoles, AZT and the alkyne are dissolved in dioxane, under nitrogen, in the presence of 5% of Ru(II)(C_5_Me_5_)(PPh_3_)Cl and heated at 100 °C for 24–48 h (Scheme 1).

**Figure 1:**
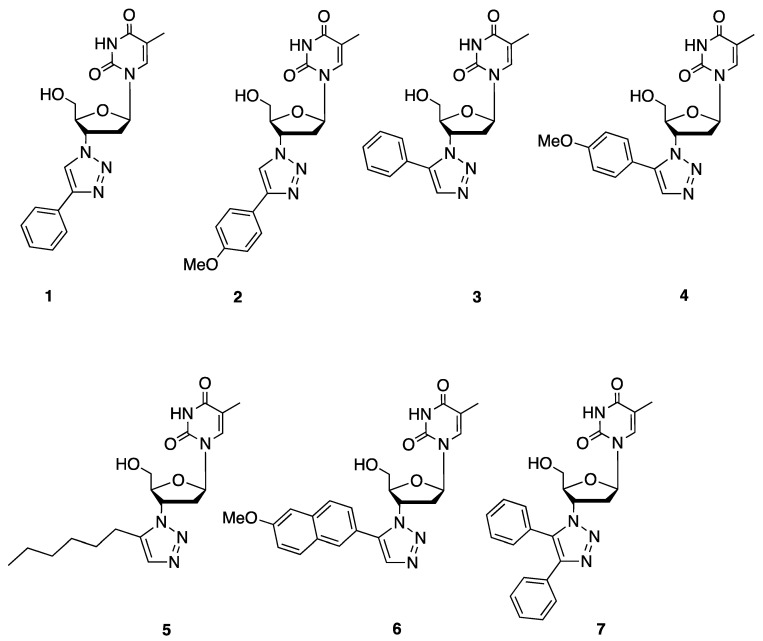
Representative 1,4- and 1,5-triazoles synthesized in this work, compounds **1**–**7**.

In both cases, purification via silica gel chromatography is required. Compounds **1**–**7** are obtained in good to moderate isolated yields (30–90%, Figure 1). 

The structure of the compounds was confirmed by NMR spectroscopy and MS spectrometry. The formation of the 1,4- and 1,5-substituted triazole is clearly indicated with the presence of a singlet, corresponding to the newly formed CH bond of the triazole ring. For 1,4-triazoles, the CH_trz_ singlet is located between 8.5–9.0 ppm, while for 1,5-triazoles, it resonates at somewhat higher field, between 7.5 and 8.0 ppm. 

Aiming to examine if the anti-viral activity is consistent with previous findings, two standard 1,4- and 1,5-phenyl derivatives, **1** and **3**, were synthesized. Additionally, compound **6**, previously reported to be the most active compound against HIV-1 [20], was synthesized for comparative purposes. We considered, based on the earlier reports on the relation between sterics and antiviral activity, that simultaneous substitution at positions 4 and 5 could be advantageous. Thus, compound **7**, bearing two phenyl groups in positions 4 and 5, was synthesized. The synthesis requires the use of the ruthenium catalyst Ru(II)(C_5_Me_5_)(PPh_3_)Cl and is not efficient with copper(I). The formation of the triazole ring in **7** is clearly indicated by the presence of two quaternary carbons at 143.4 and 134.1 ppm in the ^13^C{^1^H} NMR.

Triazoles can be easily functionalized by quaternization of N3 with suitable alkylating agent [24,25,26] to yield the corresponding salts. For the synthesis of triazolium salts, we employed exclusively methylating agents, such as methyl iodide (Alfa Aeser, Kandel, Germany) and methyl triflate (Sigma-Aldrich, St. Louis, MO, USA), for the sake of simplicity. The generated triazolium salts present, in essence, similar properties in terms of sterics, but the solubility of the compounds may vary, due to the presence of a delocalized positive charge at the triazole ring. Additionally, the quaternization can also induce variations in the ability of the molecule to undergo hydrogen bonding, known to be of importance for antiviral activity [27].

**Figure 2:**
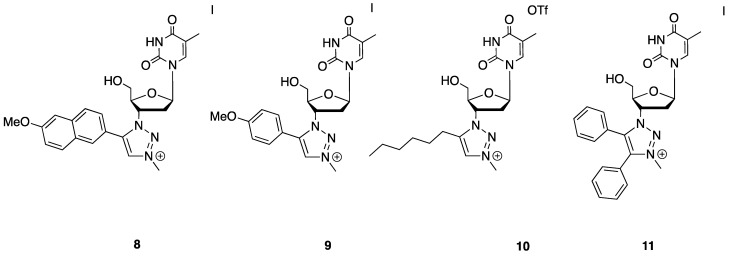
Representative methyl triazolium salts **8**–**11**.

Compounds **8**–**11** were thus synthesized and obtained in low to good yields (13–91%, Figure 2). Their formation was confirmed by NMR spectroscopy and MS. Thus, in the ^1^H NMR, and when compared to the corresponding triazoles, a significant downfield shift of circa 1 ppm is observed for the aromatic C–H of the triazole ring for compounds **8**–**10**. This feature is diagnostic of N3 quaternization due to the formation of the positive charge on the nitrogen that is delocalized within the triazole ring, inducing the deshielding of the H_trz_ proton. In addition, a new singlet is observed in the region δ 4.3–4.5 ppm indicative of the presence of the methyl group at N3 for compound **8**–**11**.

### 2.2. Antiviral Activity

**Scheme 2:**
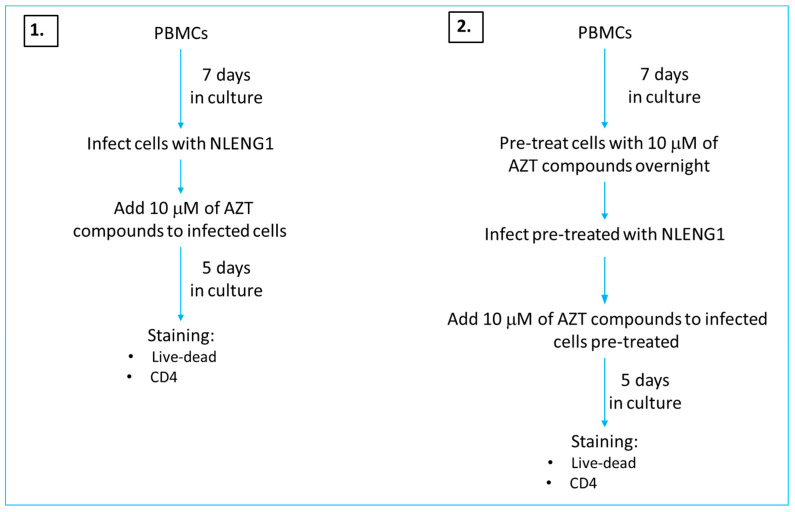
Schematic representation of methodological approach. Human peripheral blood cells were isolated from whole blood through gradient centrifugation [28,29,31]. Primary CD4^+^ T cells were infected with a GFP-tagged HIV-1 NL4.3 virus for 5 days [30]. In method 1, cells were infected with HIV-1 and then treated with AZT-derivatives. In method 2, primary T cells were pre-treated with AZT-derivatives for 18 h, then washed to remove the drugs and allow for HIV-1 infection. To restore drug treatment, 10 µM of AZT-derivative was added post-infection. At the end of the 5 days, HIV-1 replication was assessed by comparing the frequency of HIV-1 infected cells in non-treated versus AZT-derivative treated conditions by flow cytometry.

Most of our current knowledge of HIV-1 pathology has been gathered from its effects on cell lines. However, primary T cells isolated from human donors can be distinctively affected by HIV-1 infection. Thus, we determined the ability of the compounds to block HIV-1 replication in human primary CD4 T cells isolated from peripheral blood [28,29]. Thus, peripheral blood mononuclear cells (PBMCs) from healthy human donors were isolated and infected ex vivo with HIV-1 NL4.3 [30]. Two main methods were tested as depicted in Scheme 2. In the first method we tested the anti-retroviral capacity of modified AZT derivatives once HIV-1 replication had already initiated (method one). In the second method we tested the ability of the compounds to act as pre-exposure prophylaxis treatment, treating CD4 T cells with the AZT derivatives for 18 h prior to HIV-1 infection (method two). As readout, the frequency of HIV-1 infected CD4^+^ T cells was determined by intracellular flow cytometry.

**Figure 3:**
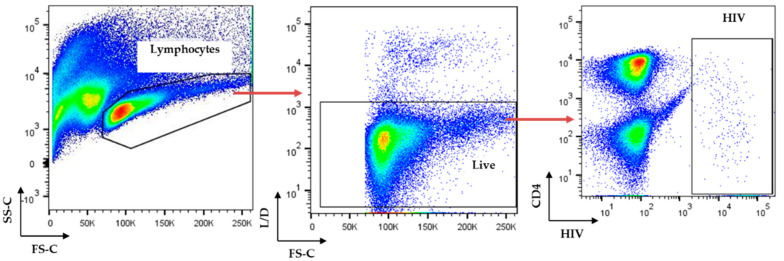
Flow cytometry gating strategy to assess the frequency of HIV-1 infected primary CD4^+^ T cells. To determine the frequency of HIV-1 infection in primary CD4^+^ T cells, peripheral mononuclear cells were sequentially gated on lymphocyte (left plot), live cells gate (middle plot), and HIV-1^+^ gate.

**Figure 4:**
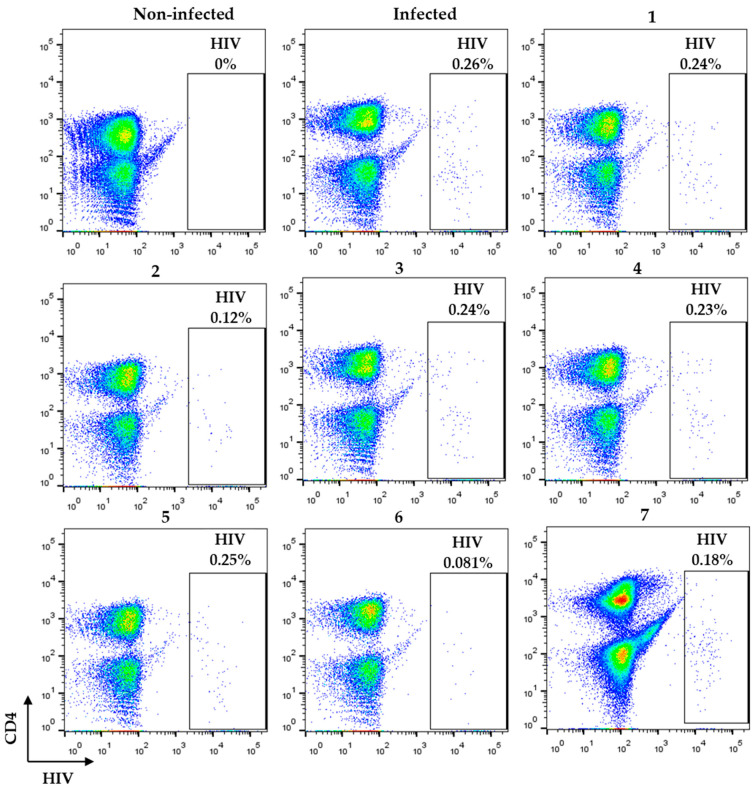
Frequency of HIV-1 infected primary CD4^+^ T cells treated with AZT-derivatives post-infection. Dot plots illustrating primary T cells HIV-1 infection levels in primary CD4^+^ T cells either left untreated (infected) or treated post-infection with AZT-derivatives (Infected+ compound).

For method one, we incubated HIV-1 infected primary CD4^+^ T cells with the live/dead fixable Viability Dye eFluor 780 (eBioscience)**.** The frequency of HIV-1 infected primary CD4^+^ T cells was determined by gating on GFP^+^ live primary CD4^+^ T cells. The percentage of HIV-1 infected cells was determined in live CD4 T cells, according to the gating strategy outlined in Figure 3. With this methodology, we found that AZT derivatives have modest effects in HIV-1 replication once the primary round of infection has been established. Indeed, only compound **2** and compound **6** were able to decrease infection levels (Figure 4). We found no significant differences between 1,4- and 1,5-triazoles, but these results corroborate the high activity of **6** as previously described [20].

**Figure 5:**
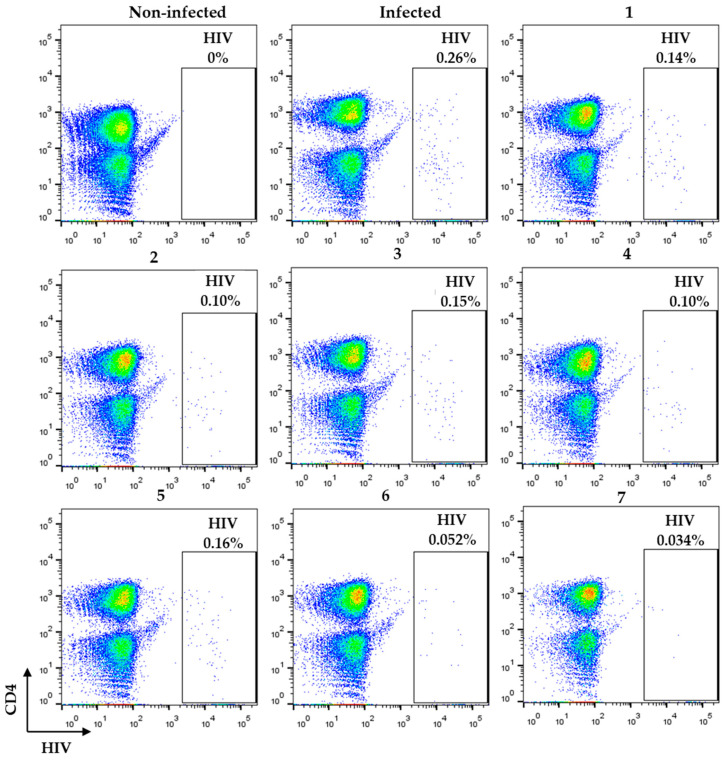
Frequency of HIV-1 infected primary CD4^+^ T cells treated with AZT-derivatives before infection. Dot plots illustrating primary T cells HIV-1 infection levels in primary CD4^+^ T cells either left untreated (infected) or treated post with AZT-derivatives (Infected+ compound).

We obtained far more promising results with AZT derivatives in our pre-exposure prophylaxis experimental model (method two, Figure 5). In this experimental approach, compounds were added for 18 h prior to infection, washed out in preparation for HIV-1 infection, and then their presence restored after infection (method two, Figure 5). Under these experimental conditions, all compounds tested were able to decrease HIV-1 infection rates by at least one half. Compounds **6** and **7** were able to clear almost all infection. The results obtained for compound **6** highlight that also for pre-exposure prophylaxis this compound is highly effective, consistent with that observed with method one. The most effective compound is compound **7**, substituted with phenyl groups at positions 4 and 5, which indicates that the combination of di-substitution and increased bulkiness can lead to an improvement in activity. 

**Figure 6:**
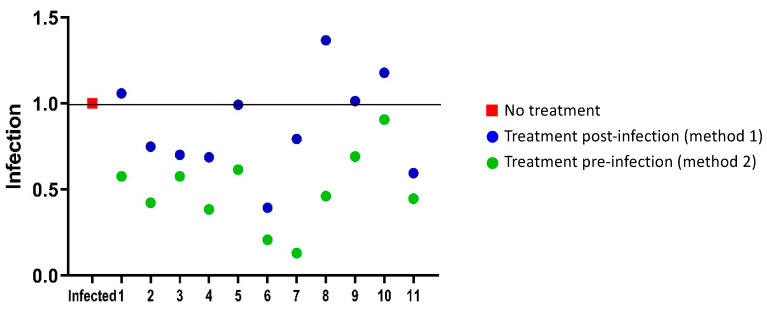
AZT-derivatives exhibit pre-exposure prophylaxis potential**.** Plotted fold-change in the frequency of HIV-1 infected CD4^+^ T cells upon treatment with different AZT-derivatives.

**Figure 7:**
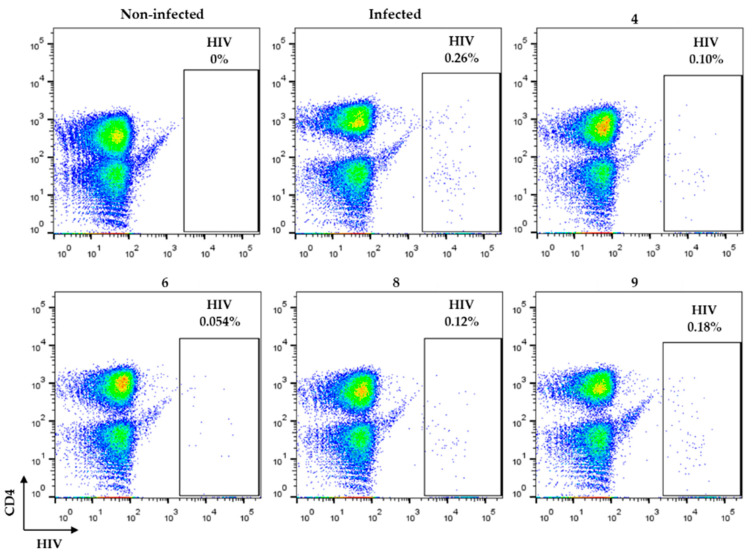
Frequency of HIV-1 infected primary CD4^+^ T cells treated with AZT-derivatives before infection. Dot plots illustrating primary T cells HIV-1 infection levels in primary CD4^+^ T cells either left untreated (infected) or treated post with AZT-derivatives (Infected + compound).

When examining the triazolium salts **8**–**11** we observed that, as was the case with their corresponding triazole precursors, compounds **8**–**11** show higher antiviral activity when method two is employed, that is, with pre-exposure of the cells to the compounds prior to infection. This difference in activity between the two methodologies is very evident for compound **8** (Figure 6). For method one, it can be observed that quaternization decreases antiviral activity for complexes **8**–**10**, when compared to the triazole precursors **4**–**6**, with the only exception being compound **11,** which shows a slightly higher activity than that found for triazole **7**. For method two, the decrease of antiviral activity upon quaternization is observed for all cases, showing that the formation of the salt is detrimental and does not foster the activity. Indeed, when compared to the triazole precursors, the triazolium salts showed a decreased activity in all cases, as can been seen, for example, for complexes **6** versus **8** and **4** versus **9** (Figure 7). Triazoles are more lipophilic than their corresponding triazolium salts, which is an indication that triazoles are more prone to cross the cell membrane, which could be related to their higher activity. 

Thus, it can be concluded that, in general, triazolium salts were less effective than their triazole counterparts, showing, that alkylation of the triazole derivatives of AZT tends to decrease antiviral activity. 

## 3. Conclusions

Despite the efficacy of AZT, the search for other HIV-1 reverse transcription inhibitors has continued due to AZT poor bioavailability at the sites of HIV-1 replication (lymphoid organs), the emergence of HIV-1 resistant strains, and secondary effects. We described a series of AZT derivatives modified with triazoles that are particularly efficient if provided prior to HIV-1 infection. Under these conditions, compounds were able to decrease infection and two of them, compounds **6** and **7**, were able to clear almost all the infection. The high activity found for compound **7** is indicative that substitution with aromatic groups at positions **4** and **5** of the triazole ring leads to an improvement of the antiviral activity. Lastly, we found that triazolium salts are less effective that their triazole precursors, showing, in general, a decrease in antiviral activity. Overall, these results suggest triazoles based on AZT could play an important role not only in HIV-1 treatment but also in pre-exposure prophylaxis therapies. 

## 4. Materials and Methods

The syntheses of complexes were carried out under an inert atmosphere of N_2_ using Schlenk techniques. 3′-Azido-3′-deoxythymidine (Carbosynth, Berkshire, UK), ethynylanisole (TCI, Tokio, Japan), CuSO_4_ (Alfa Aesar, Karlsruhe, Germany), and sodium ascorbate (Sigma-Aldrich, MO, USA),Cp*RuCl(PPh_3_)_2_ (Sigma-Aldrich, MO, USA), 1-octyne (TCI, Tokio, Japan) and diphenylacetylene (Sigma-Aldrich, MO, USA) were used without further purification. *tert*-Butanol, Et_2_O, DCM, MeOH, dioxane, pentane, DMF and acetone were commercial solvents of analytical grade used as received. All ^1^H and ^13^C{^1^H} NMR spectra (see Supplementary) were recorded at room temperature on Bruker spectrometers (400 MHz). Chemical shifts are reported as δ-values in ppm relative to the DMSO-*d*_6_ solvent peaks (δH: 2.50; δC: 39.52). Compounds **1**, **3**, and **6** were synthesized according to previously reported procedures [20]. Mass Spectroscopy measurements were obtained by the UniMass Laboratory at Instituto de Tecnologia Química e Biológica, Portugal.

Compound **2**: A suspension of 3′-azido-3′-deoxythymidine (100 mg, 0.375 mmol), ethynylanisole (60 mg, 0.450 mmol), CuSO_4_ (3.5 mg, 0.022 mmol), and sodium ascorbate (45 mg, 0.225 mmol) in *tert*-butanol (5 mL) was stirred at 100 °C for 2 h. After cooling, the solid residue was filtered-off and washed with Et_2_O. The solid residue was purified by silica gel chromatography (DCM:MeOH 10:1 *v*/*v*). Compound **2** was obtained as a white powder (50 mg, 33%). ^1^H NMR (400 MHz, DMSO-*d_6_*) δ 11.37 (1H, s, NH), 8.67 (1H, s, H_Trz_), 7.85 (1H, s, H-6), 7.79 (2H, d, ^3^*J*_H(*meta*),H(*ortho*)_ = 8.8 Hz, MeOPh−2×C*H_meta_*), 7.04 (2H, d, ^3^*J*_H(*ortho*),H(*meta*)_ = 8.8 Hz, MeOPh−2×C*H_ortho_*), 6.45 (1H, dd, ^3^*J*_H-1′,H-2′a_ = 6.4 Hz, ^3^*J*_H-1′,H-2′b_ = 6.8 Hz, H-1′), 5.45–5.34 (1H, m, H-3′), 5.31 (1H, dd, ^3^*J*_5′OH,H-5′a_ = 5.2 Hz, ^3^*J*_5′OH,H-5′b_ = 5.6 Hz, 5′-OH), 4.33–4.22 (1H, m, H-4′), 3.80 (3H, s, MeOPh−C*H*_3_), 3.77–3.58 (2H, m, H-5′), 2.88–2.62 (2H, m, H-2′), 1.83 (3H, s, 5-C*H*_3_). ^13^C{^1^H}NMR (400 MHz, DMSO-*d_6_*) δ 163.8 (C-4), 159.1 (Trz−C_quat_), 150.5 (C-2), 146.5 (MeOPh−C^1^), 136.3 (C-6), 126.5 (MeOPh−2×C*_meta_*), 123.2 (MeOPh−C*_para_*), 120.0 (Trz−C*H*), 114.4 (MeOPh−2×C*_ortho_*), 109.7 (C-5), 84.4 (C-4′), 83.9 (C-1′), 60.8 (C-5′), 59.3 (C-3′), 55.2 (MeOPh−O*C*H_3_), 37.1 (C-2′), 12.3 (5-CH_3_). HRMS-ESI (*m*/*z*): [M+H]^+^ calc. for C_19_H_21_N_5_O_5_ 400.1615. Found 400.1612.

Compound **4**: 3′-azido-3′-deoxythymidine (100 mg, 0.37 mmol), ethynylanisole (97 mg, 0.75 mmol), and Cp*RuCl(PPh_3_)_2_ (0.05 eq.) were dissolved in dioxane (5 mL) under N_2_ atmosphere, the resulting mixture was stirred at 80 °C for 48 h. After cooling, the solvent was removed under reduced pressure and the solid residue was purified by silica gel chromatography (DCM:MeOH 10:1 *v*/*v*). Compound **4** was obtained as a brown powder (47 mg, 31%). ^1^H NMR (400 MHz, DMSO-*d_6_*) δ 11.36 (1H, s, NH), 7.85 (1H, s, H_Trz_), 7.78 (1H, s, H-6), 7.46 (2H, d, ^3^*J*_H(*meta*),H(*ortho*)_ = 8.8 Hz, MeOPh−2×C*H_meta_*), 7.11 (2H, d, ^3^*J*_H(*ortho*),H(*meta*)_ = 8.8 Hz, MeOPh−2×C*H_ortho_*), 6.56 (1H, dd, ^3^*J*_H-1′,H-2′a_ = 6.4 Hz, ^3^*J*_H-1′,H-2′b_ = 7.6 Hz, H-1′), 5.13 (1H, ddd, ^3^*J*_H-3′,H-2′a_ ≈ 3.0 Hz, ^3^*J*_H-3′,H-2′b_ = 3.4 Hz, ^3^*J*_H-3′,H-4′_ = 7.6 Hz, H-3′), 4.42-4.31 (1H, m, H-4′), 3.83 (3H, s, MeOPh−C*H*_3_), 3.60 (1H, dd, ^3^*J*_H-5′a,H-4′_ ≈ 4 Hz, ^2^*J*_H-5′a,H-5′b_ = 12.0 Hz, H-5′a), 3.49 (1H, dd, ^3^*J*_H-5′b,H-4′_ = 3.2 Hz, ^2^*J*_H-5′b,H-5′a_ = 12.0 Hz, H-5′b), 2.65–2.55 (2H, m, H-2′), 1.78 (3H, s, 5-C*H*_3_). ^13^C{^1^H}NMR (400 MHz, DMSO-*d_6_*) δ 163.8 (C-4), 160.2 (MeOPh−C*_para_*), 150.5 (C-2), 137.7 (MeOPh−C^1′’^), 136.1 (C-6), 132.7 (Trz−*C*H), 130.7 (MeOPh−2×C*_meta_*), 118.4 (Trz−C_quat_), 114.6 (MeOPh−2×C*_ortho_*), 109.7 (C-5), 84.9 (C-4′), 84.6 (C-1′), 61.4 (C-5′), 58.2 (C-3′), 55.4 (MeOPh−O*C*H_3_), 37.8 (C-2′), 12.3 (5-*C*H_3_). HRMS-ESI (*m*/*z*): [M+H]^+^ calc. for C_19_H_21_N_5_O_5_ 400.1615. Found 400.1611.

Compound **5**: A mixture of 3′-azido-3′-deoxythymidine (100 mg, 0.375 mmol), 1-octyne (110 μL, 0.748 mmol), and Cp*RuCl(PPh_3_)_2_ (15 mg, 0.05 eq.) was dissolved in dioxane (5 mL) under N_2_ atmosphere. The resulting reaction mixture was stirred at 80 °C for 48 h. After cooling, the solvent was removed under vacuum on a rotary evaporator. The solid residue was purified by silica gel chromatography (DCM:MeOH 9:1 *v*/*v*). Compound **5** was obtained as a white powder (123 mg, 87%). ^1^H NMR (400 MHz, DMSO-*d_6_*) δ 11.35 (1H, s, NH), 7.80 (1H, s, H-6), 7.57 (1H, s, H_Trz_), 6.51 (1H, t, ^3^*J*_H-1′,H-2′_ = 6.8 Hz, H-1′), 5.33 (1H, dd, ^3^*J*_5′OH,H-5′a_ = 6.0 Hz, ^3^*J*_5′OH,H-5′b_ = 6.4 Hz, 5′-OH), 5.11 (1H, dt, ^3^*J*_H-3′,H-4′_ = 5.2 Hz, ^3^*J*_H-3′,H-2′_ = 6.4 Hz, H-3′), 4.26-4.15 (1H, m, H-4′), 3.75–3.59 (2H, m, H-5′), 2.69 (2H, t, ^3^*J*_H-1′’,H-2′’_ = 7.8 Hz, Hexyl−C^1′’^*H*_2_), 2.61 (2H, dd, ^3^*J*_H-2′,H-3′_ = 6.4 Hz, ^3^*J*_H-2′,H-1′_ = 6.8 Hz, H-2′), 1.81 (3H, s, 5-C*H*_3_), 1.60 (2H, quint, ^3^*J*_H-2′’,H-1′’_ = ^3^*J*_H-2′’,H-3′’_ = 7.8 Hz, Hexyl−C^2′’^*H*_2_), 1.40–1.22 (6H, m, Hexyl−C^3′’^*H*_2_+C^4′’^*H*_2_+C^5′’^*H*_2_), 0.86 (3H, t, ^3^*J*_H-6′’,H-5′’_ = 6.8 Hz, Hexyl−C^6′’^*H*_3_). ^13^C{^1^H}NMR (400 MHz, DMSO-*d_6_*) δ 163.8 (C-4), 150.5 (C-2), 137.9 (Trz−*C*_quat_), 136.2 (C-6), 131.7 (Trz−*C*H), 109.7 (C-5), 84.9 (C-4′), 84.5 (C-1′), 61.2 (C-5′), 57.0 (C-3′), 37.2 (C-2′), 30.9, 28.3, 27.9, 22.5, 22.4 (Hexyl−5×*C*H_2_), 14.0 (Hexyl−*C*^6′’^H_3_), 12.3 (5-*C*H_3_). HRMS-ESI (*m*/*z*): [M+H]^+^ calc. for C_18_H_27_N_5_O_4_ 378.2135. Found 378.2130.

Compound **7**: A mixture of 3′-azido-3′-deoxythymidine (150 mg, 0.568 mmol), diphenylacetylene (202 mg, 1.14 mmol), and Cp*RuCl(PPh_3_)_2_ (22 mg, 0.05 eq.) was dissolved in dioxane (3 mL) under N_2_ atmosphere. The resulting mixture was stirred at 60 °C for 48 h. After cooling, Et_2_O was added to the reaction mixture and a dark green solid precipitated. The solid was filtered and pentane was then added to the filtrate, precipitating a light green/white solid. This solid was filtered and washed with Et_2_O and pentane before it was let to dry. Compound **7** was obtained as a light green/white powder (146 mg, 58%). ^1^H NMR (400 MHz, DMSO-*d_6_*) δ 11.36 (1H, s, NH), 7.76 (1H, s, H-6), 7.65-7.56 (3H, m, Ph−3×C*H*), 7.53-7.41 (4H, m, Ph−4×C*H_ortho_*), 7.36-7.23 (3H, m, Ph−3×C*H*), 6.58 (1H, dd, ^3^*J*_H-1′,H-2′a_ = 6.0 Hz, ^3^*J*_H-1′,H-2′b_ = 8.4 Hz, H-1′), 5.31 (1H, t, ^3^*J*_5′OH,H-5′a_ = ^3^*J*_5′OH,H-5′b_ = 4.8 Hz, 5′-OH), 4.87 (1H, dt, ^3^*J*_H-3′,H-2′a_ = ^3^*J*_H-3′,H-2′b_ = 2.4 Hz, ^3^*J*_H-3′,H-4′_ = 8.4 Hz, H-3′), 4.47-4.37 (1H, m, H-4′), 3.55 (1H, ddd, ^3^*J*_H-5′a,H-4′_ = 4.0 Hz, ^3^*J*_H-5′a,OH-5′_ = 4.8 Hz, ^2^*J*_H-5′a,H-5′b_ = 12.0 Hz, H-5′a), 3.38 (1H, ddd, ^3^*J*_H-5′b,H-4′_ = 4.0 Hz, ^3^*J*_H-5′b,OH-5′_ = 4.8 Hz, ^2^*J*_H-5′b,H-5′a_ = 12.0 Hz, H-5′b), 2.71 (1H, ddd, ^3^*J*_H-2′a,H-3′_ = 2.4 Hz, ^3^*J*_H-2′a,H-1′_ = 6.0 Hz, ^3^*J*_H-2′a,H-2′b_ = 13.8 Hz, H-2′a), 2.53–2.44* (1H, m, H-2′b), 1.76 (3H, s, 5-C*H*_3_). ^13^C{^1^H}NMR (400 MHz, DMSO-*d_6_*) δ 163.7 (C-4), 150.5 (C-2), 143.4 (Trz−C_quat_), 136.1 (C-6), 134.1 (Trz−C_quat_), 130.7 (Ph−C_quat_), 130.4, 130.1, 129.5, 128.6, 127.8 (Ph−C*H*), 127.1 (Ph−C_quat_), 126.3 (Ph−C*H*), 109.7 (C-5), 84.8 (C-4′), 84.7 (C-1′), 61.4 (C-5′), 58.6 (C-3′), 37.4 (C-2′), 12.3 (5-CH_3_). * Determined by HSQC. HRMS-ESI (*m*/*z*): [M+H]^+^ calc. for C_24_H_23_N_5_O_4_ 446.1822. Found 446.1823.

Compound **8**: Compound **6** (37.4 mg, 8.32 × 10^−3^ mmol) was dissolved in DMF (3 mL) under N_2_ atmosphere and methyl iodide (17.6 μL, 2.83 × 10^−2^ mmol) was added. The resulting mixture was stirred at 100 °C for 24 h. After cooling to room temperature, Et_2_O was added and an orange oil precipitated. The oily product was continuously washed with Et_2_O until a solid formed, which was then filtered-off and dried under vacuum (59.9 mg, 91%). ^1^H NMR (400 MHz, DMSO-*d_6_*) δ 11.39 (1H, s, NH), 9.17 (1H, s, H_Trz_), 8.18 (1H, d, ^4^*J*_H-10′’,H-2′’_ = 1.8 Hz, MeONapht. H-10′’), 8.11 (1H, d, ^3^*J*_H-3′’,H-2′’_ = 8.4 Hz, MeONapht. H-3′’), 8.02 (1H, d, ^3^*J*_H-8′’,H-7′’_ = 9.2 Hz, MeONapht. H-8′’), 7.76 (1H, d, ^4^*J*_H-6,5-CH3_ = 1.2 Hz, H-6), 7.66 (1H, dd, ^4^*J*_H-2′’,H-10′’_ = 1.8 Hz, ^3^*J*_H-2′’,H-3′’_ = 8.4 Hz, MeONapht. H-2′’), 7.51 (1H, dd, ^4^*J*_H-5′’,H-7′’_ = 2.8 Hz, MeONapht. H-5′’), 7.35 (1H, dd, ^4^*J*_H-7′’,H-5′’_ = 2.8 Hz, ^3^*J*_H-7′’,H-8′’_ = 9.2 Hz, MeONapht. H-7′’), 6.47 (1H, dd, ^3^*J*_H-1′,H-2′a_ = 6.4 Hz, ^3^*J*_H-1′,H-2′b_ = 8.0 Hz, H-1′), 5.48 (1H, ddd, ^3^*J*_H-3′,H-2′a_ ≈ ^3^*J*_H-3′,H-2′b_ = 2.8 Hz, ^3^*J*_H-3′,H-4′_ = 8.8 Hz, H-3′), 5.34 (1H, t, ^3^*J*_5′OH,H-5′a_ = ^3^*J*_5′OH,H-5′b_ = 4.8 Hz, 5′-OH), 4.60–4.53 (1H, m, H-4′), 4.45 (3H, s, N_Trz_−C*H*_3_), 3.94 (3H, s, MeONapht. C*H*_3_), 3.61 (1H, dt, ^3^*J*_H-5′a,H-4′_ = ^3^*J*_H-5′a_,_5′OH_ = 4.8 Hz, ^2^*J*_H-5′a,H-5′b_ = 12.0 Hz, H-5′a), 3.54 (1H, dt, ^3^*J*_H-5′b,H-4′_ = ^3^*J* _H-5′b_,_5′OH_ = 4.8 Hz, ^2^*J*_H-5′b,H-5′a_ = 12.0 Hz, H-5′b), 2.90 (1H, dd, ^3^*J*_H-2′a,H-3′_ = 2.8 Hz, ^3^*J*_H-2′a,H-1′_ = 6.4 Hz, ^2^*J*_H-2′a,H-2′b_ = 14.4 Hz, H-2′a), 2.74 (1H, dd, ^3^*J*_H-2′b,H-3′_ = 2.4 Hz, ^3^*J*_H-2′b,H-1′_ ≈ 8.8 Hz, ^2^*J*_H-2′b,H-2′a_ = 14.4 Hz, H-2′b), 1.76 (3H, d, ^4^*J*_5-CH3,H-6_ = 1.2 Hz, 5-C*H*_3_). ^13^C{^1^H}NMR (400 MHz, DMSO-*d_6_*) δ 163.7 (C-4), 159.1 (MeONapht.−*C*_quart_OMe), 150.5 (C-2), 142.9 (MeONapht.−*C*^1′’^_quat_), 136.0 (C-6), 135.5 (MeONapht.−*C*^4′’^_quat_), 130.3 (MeONapht.−*C*^8′’^H), 130.2 (Trz−C*H* + MeONapht.−*C*^10′’^H), 128.1 (MeONapht.−*C*^3′’^H), 127.7 (MeONapht.−*C*^9′’^_quat_), 126.3 (MeONapht.−*C*^2′’^H), 120.3 (MeONapht.−*C*^7′’^H), 117.0 (Trz−C_quat_), 109.9 (C-5), 106.1 (MeONapht.−*C*^5′’^H), 84.6 (C-1′), 84.2 (C-4′), 62.1 (C-3′), 61.2 (C-5′), 55.5 (MeONapht.−O*C*H_3_), 40.5 (Trz−N*C*H_3_), 37.3 (C-2′), 12.3 (5-*C*H_3_). HRMS-ESI (*m*/*z*): [M]^+^ calc. for C_24_H_26_N_5_O_5_ 464.1928. Found 464.1925.

Compound **9:** Compound **4** (42.0 mg, 0.105 mmol) was dissolved in DMF (2 mL) and methyl iodide (65 μL, 1.0 mmol) was added. The resulting mixture was stirred at 100 °C for 24 h. After cooling to room temperature, Et_2_O was added and an orange oil precipitated. The oily product was dissolved in acetone and continuously washed with Et_2_O until a solid formed, which was then filtered-off and dried under vacuum (44 mg, 78%). ^1^H NMR (400 MHz, DMSO-*d_6_*) δ 11.38 (1H, s, NH), 9.05 (1H, s, H_Trz_), 7.76 (1H, s, H-6), 7.59 (2H, d, ^3^*J*_H(*meta*),H(*ortho*)_ = 8.8 Hz, MeOPh−2×C*H_meta_*), 7.23 (2H, d, ^3^*J*_H(*ortho*),H(*meta*)_ = 8.8 Hz, MeOPh−2×C*H_ortho_*), 6.43 (1H, dd, ^3^*J*_H-1′,H-2′a_ = 6.3 Hz, ^3^*J*_H-1′,H-2′b_ = 8.4 Hz, H-1′), 5.34 (1H, t, ^3^*J*_5′OH,H-5′a_ = ^3^*J*_5′OH,H-5′b_ = 5.0 Hz, 5′-OH), 5.40-5.34* (1H, m, H-3′), 4.55-4.49 (1H, m, H-4′), 4.41 (3H, s, Trz−C*H*_3_), 3.87 (3H, s, MeOPh−C*H*_3_), 3.60 (1H, dt, ^3^*J*_H-5′a,5′OH_ ≈ ^3^*J*_H-5′a,H-4′_ ≈ 5.0 Hz, ^2^*J*_H-5′a,H-5′b_ = 11.7 Hz, H-5′a), 3.53 (1H, dd, ^3^*J*_H-5′b,5′OH_ ≈ ^3^*J*_H-5′b,H-4′_ ≈ 5.0 Hz, ^2^*J*_H-5′b,H-5′a_ = 11.7 Hz, H-5′b), 2.85 (1H, ddd, ^3^*J*_H-2′a,H-3′_ = 2.2 Hz, ^3^*J*_H-2′a,H-1′_ = 6.3 Hz, ^2^*J*_H-5′b,H-5′a_ = 14.5 Hz, H-2′b), 2.72-2.63 (1H, m, H-2′b), 1.78 (3H, s, 5-C*H*_3_). ^13^C{^1^H}NMR (400 MHz, DMSO-*d_6_*) δ 163.7 (C-4), 160.2 (MeOPh−C*_para_*), 150.5 (C-2), 137.7 (MeOPh−*C*^1′’^_quat_), 136.0 (C-6), 131.6 (MeOPh−2×C*_meta_*), 129.9 (Trz−*C*H), 115.1 (MeOPh−2×C*_ortho_*), 114.1 (Trz−*C*_quat_), 109.9 (C-5), 84.6 (C-1′), 84.1 (C-4′), 61.8 (C-3′), 61.2 (C-5′), 55.6 (MeOPh−O*C*H_3_), 40.7 (Trz−N*C*H_3_), 37.2 (C-2′), 12.3 (5-*C*H_3_). HRMS-ESI (*m*/*z*): [M]^+^ calc. for C_20_H_24_N_5_O_5_ 414.1771. Found 414.1777.

Compound **10**: Compound **5** (110.3 mg, 0.3035 mmol) was dissolved in DMF (2.5 mL) under N_2_ atmosphere and methyl triflate (66 μL, 0.60 mmol) was added. The resulting mixture was stirred overnight at 100 °C. After cooling to room temperature, Et_2_O was added and an orange oil precipitated. The oily product was continuously washed with Et_2_O and dried under vacuum. Compound **11** was obtained as an oily orange solid (21.9 mg, 13%). ^1^H NMR (400 MHz, DMSO-*d_6_*) δ 11.39 (1H, s, NH), 8.77 (1H, s, H_Trz_), 7.78 (1H, d, ^4^*J*_H-6,5-CH3_ = 1.2 Hz, H-6), 6.41 (1H, t, ^3^*J*_H-1′,H-2′_ = 6.8 Hz, H-1′), 5.53-5.45 (1H, m, H-3′), 5.53 (1H, t, ^3^*J*_5′OH,H-5′a_ = ^3^*J*_5′OH,H-5′b_ = 5.0 Hz, 5′-OH), 4.39-4.33 (1H, m, H-4′), 4.31 (3H, s, N-C*H*_3_), 3.78-3.63 (2H, m, H-5′), 2.90 (2H, t, ^3^*J*_H-1′’,H-2′’_ = 7.6 Hz, Hexyl−C^1′’^*H*_2_), 2.76 (2H, t, ^3^*J*_H-2′,H-1′_ = ^3^*J*_H-2′,H-3′_ = 6.8 Hz, H-2′), 1.81 (3H, d, ^4^*J*_5-CH3,H-6_ = 1.2 Hz, 5-C*H*_3_), 1.67 (2H, quint, ^3^*J*_H-2′’,H-1′’_ = ^3^*J*_H-2′’,H-3′’_ = 7.6 Hz, Hexyl−C^2′’^*H*_2_), 1.44-1.35 (2H, m, Hexyl−C^3′’^*H*_2_), 1.35-1.26 (4H, m, Hexyl−C^4′’^*H*_2_+C^5′’^*H*_2_), 0.88 (3H, t, ^3^*J*_H-6′’,H-5′’_ = 7.0 Hz, Hexyl−C^6′’^*H*_3_). ^13^C{^1^H}NMR (400 MHz, DMSO-*d_6_*) δ 163.7 (C-4), 150.4 (C-2), 144.4 (Trz−*C*_quat_), 136.0 (C-6), 129.3 (Trz−*C*H), 109.5 (C-5), 84.4 (C-4′), 84.0 (C-1′), 61.0 (C-5′), 60.9 (C-3′), 40.2 (N-*C*H_3_), 36.7 (C-2′), 30.8 (Hexyl−*C*H_2_), 27.9 (Hexyl−*C*^3′’^H_2_), 26.5 (Hexyl−*C*^2′’^H_2_), 22.4 (Hexyl−*C*^1′’^H_2_), 21.9 (Hexyl−*C*^3′’^H_2_), 13.9 (Hexyl−*C*^6′’^H_3_), 12.3 (5-*C*H_3_). HRMS-ESI (*m*/*z*): [M]^+^ calc. for C_19_H_30_N_5_O_4_ 392.2292. Found 392.2291.

Compound **11**: Compound **7** (100 mg, 0.246 mmol) was dissolved in DMF (3 mL) under N_2_ atmosphere and methyl iodide (23 μL, 0.449 mmol) was added. The resulting mixture was stirred at 100 °C for 24 h. After cooling to room temperature, Et_2_O was added and an orange oil precipitated. The oily product was continuously washed with Et_2_O until a solid formed, which was then filtered-off and dried under vacuum (66.9 mg, 51%). ^1^H NMR (400 MHz, DMSO-*d_6_*) δ 11.40 (1H, s, NH), 7.75 (1H, d, ^4^*J*_H-6,5-CH3_ = 1.2 Hz, H-6), 7.64–7.46 (10H, m, Ph−10×C*H*), 6.49 (1H, dd, ^3^*J*_H-1′,H-2′a_ = 6.2 Hz, ^3^*J*_H-1′,H-2′b_ = 8.6 Hz, H-1′), 5.37 (1H, t, ^3^*J*_5′OH,H-5′a_ = ^3^*J*_5′OH,H-5′b_ = 4.8 Hz, 5′-OH), 5.25 (1H, dt, ^3^*J*_H-3′,H-2′a_ = ^3^*J*_H-3′,H-2′b_ = 2.8 Hz, ^3^*J*_H-3′,H-4′_ = 8.4 Hz, H-3′), 4.63-4.55 (1H, m, H-4′), 4.30 (1H, s, NC*H*_3_), 3.65-3.57 (1H, m, H-5′a), 3.55-3.48* (1H, m, H-5′b), 2.90 (1H, ddd, ^3^*J*_H-2′a,H-3′_ ≈ 2.8 Hz, ^3^*J*_H-2′a,H-1′_ ≈ 6.2 Hz, ^2^*J*_H-2′a,H-2′b_ = 14.4 Hz, H-2′a), 2.68 (1H, ddd, ^3^*J*_H-2′b,H-3′_ = ^3^*J*_H-2′b,H-1′_ ≈ 6.2 Hz, ^2^*J*_H-2′b,H-2′a_ = 14.4 Hz, H-2′b), 1.77 (3H, d, ^4^*J*_5-CH3,H-6_ = 1.2 Hz, 5-C*H*_3_). ^13^C{^1^H}NMR (400 MHz, DMSO-*d_6_*) δ 163.7 (C-4), 150.5 (C-2), 140.3 (Trz−C^4′’^), 140.2 (Ph−C_quat_), 136.0 (C-6), 131.6 (Ph−C*H*), 131.5 (Ph−C_quat_), 130.6, 130.1, 129.5, 129.4 (Ph−C*H*), 122.4 (Trz−C^5′’^), 109.9 (C-5), 84.7 (C-1′), 84.1 (C-4′), 61.2 (C-5′), 58.6 (C-3′), 38.6* (N*C*H_3_), 36.9 (C-2′), 12.3 (5-CH_3_). *Determined by HSQC. HRMS-ESI (*m*/*z*): [M]^+^ calc. for C_25_H_23_N_5_O_4_ 460.1979. Found 460.1981.

## Supplementary Materials

NMR spectra for all the compounds are available online.
