# Supplementary material for: Development of Triazoles and Triazolium Salts Based on AZT and Their Anti-Viral Activity against HIV-1
Source: Molecules. 2021 Nov 6;26(21):6720. doi: 10.3390/molecules26216720 (PMC8588071; doi:10.3390/molecules26216720)
Supplement: Supplementary file 1 [file molecules-26-06720-s001.zip › molecules-1437781-supplementary.pdf]

Supporting Information

Development of Triazoles and Triazolium Salts based on AZT and their Anti-Viral Activity Against HIV-1

Daniel Machado de Alencar [1], Juliana Gonçalves[2], Andreia Vieira[1], Sofia A. Cerqueira[2], Cruz Sebastião[2], Maria Inês P.S. Leitão, Giulia Francescato, Paola Antenori, Helena Soares[2,*] and Ana Petronilho[1,*]

[1] ITQB – Instituto de Tecnologia Química e Biológica, Universidade Nova de Lisboa, Estação Agronómica Nacional, Oeiras, Portugal.

[2] Human Immunobiology and Pathogenesis Laboratory, Chronic Diseases Research Center, NOVA Medical School, NOVA University of Lisbon, 1150-082 Lisbon, Portugal

* Correspondence: ana.petronilho@itqb.unl.pt; helena.soares@nms.unl.pt

NMR Spectra

Compound 2

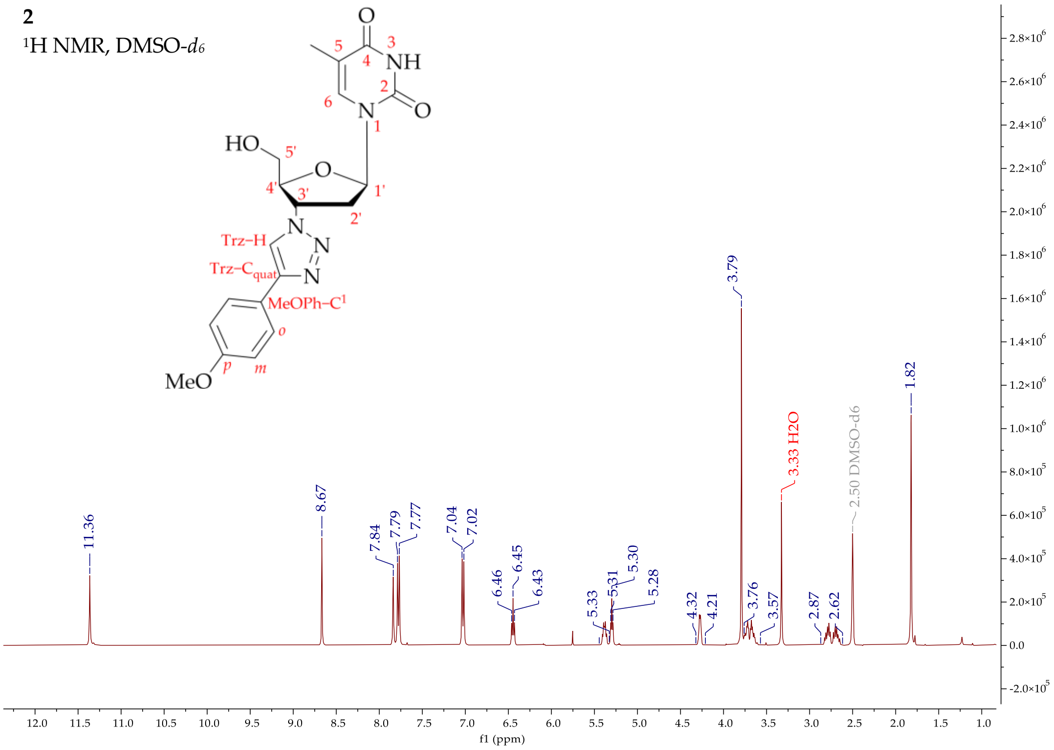

Figure S1. ^{1}H NMR spectrum of compound **2** recorded in DMSO-*d*6.

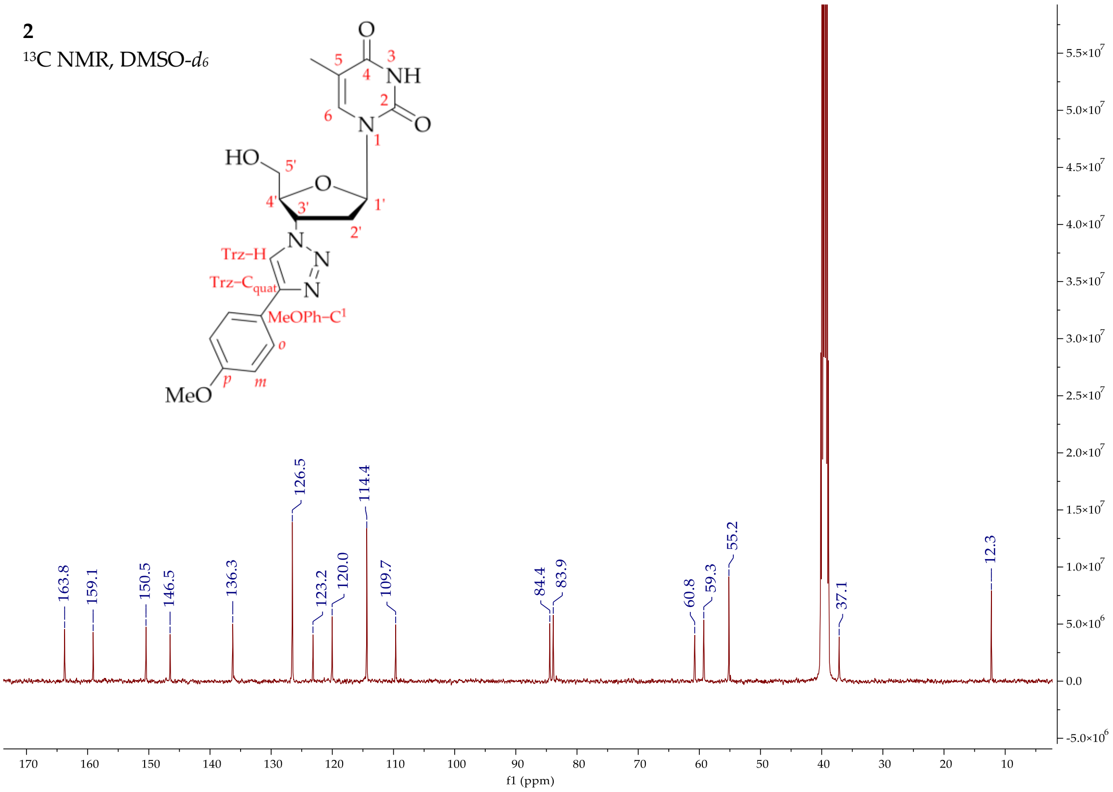

Figure S2. $^{13}C\{^{1}H\}$ NMR spectrum of compound **2** recorded in DMSO-*d*6.

Compound 4

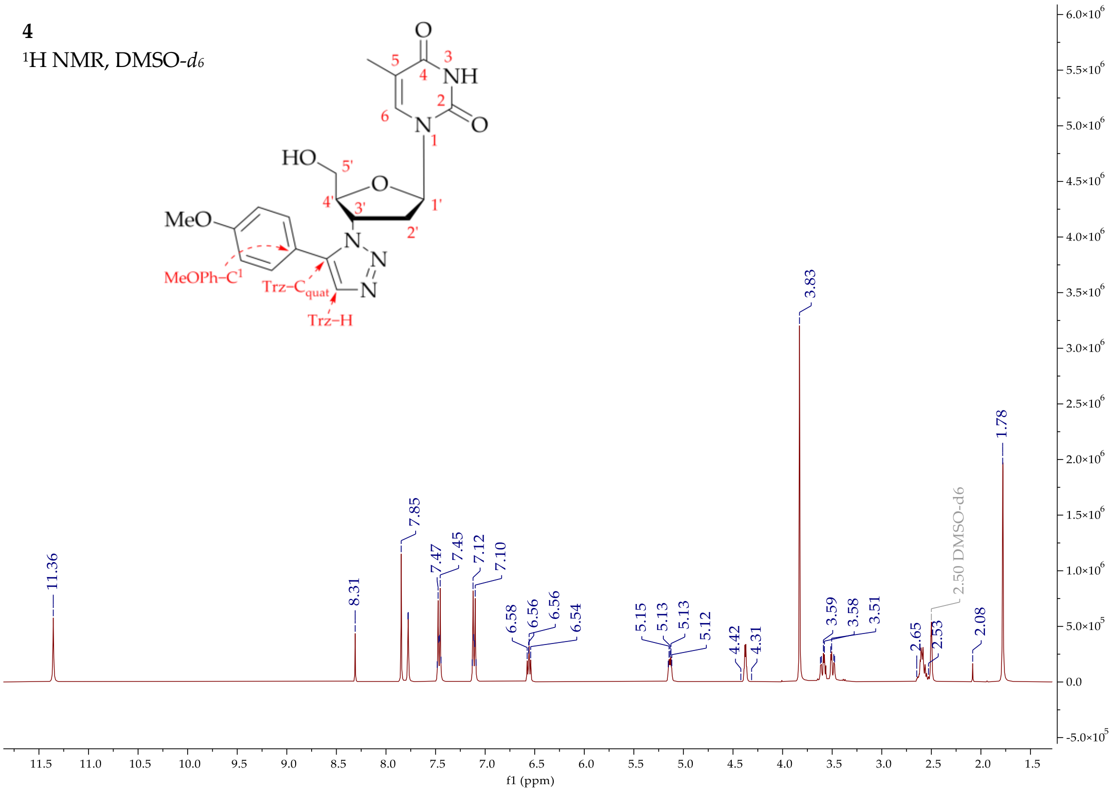

Figure S3. ^{1}H NMR spectrum of compound **4** recorded in DMSO-*d*$_6$.

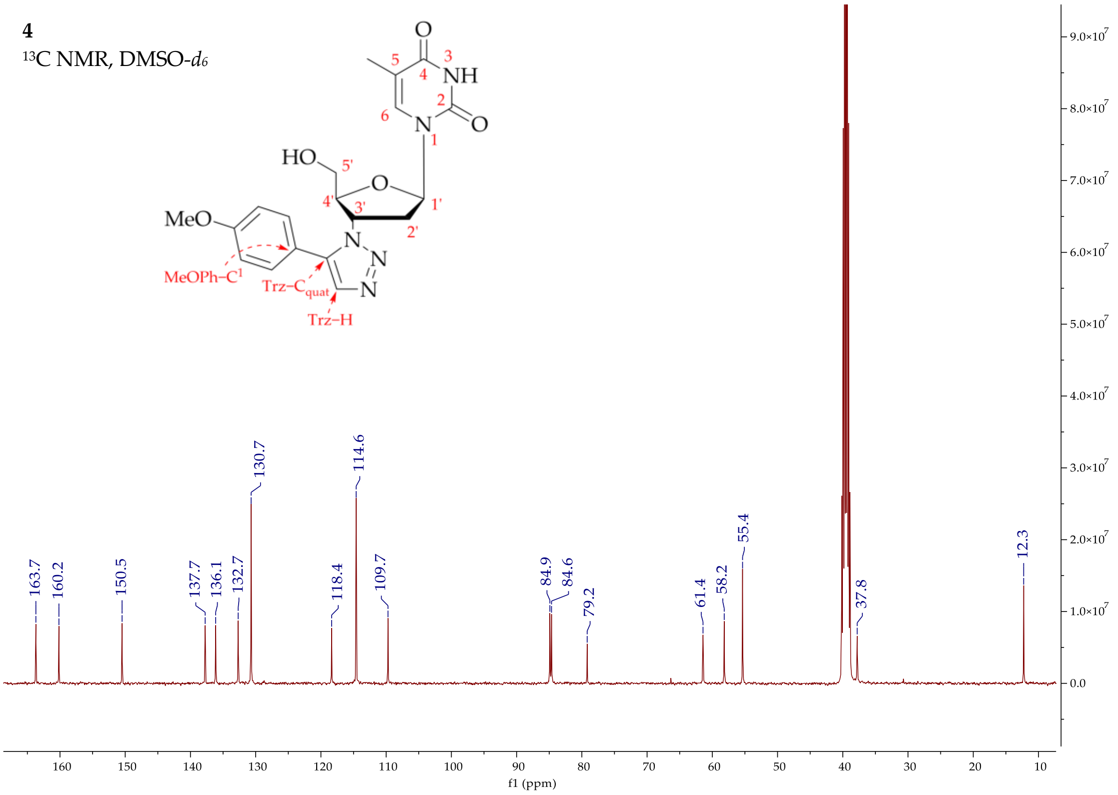

Figure S4. ^{13}C NMR spectrum of compound **4** recorded in DMSO-*d*$_6$.

Compound 5

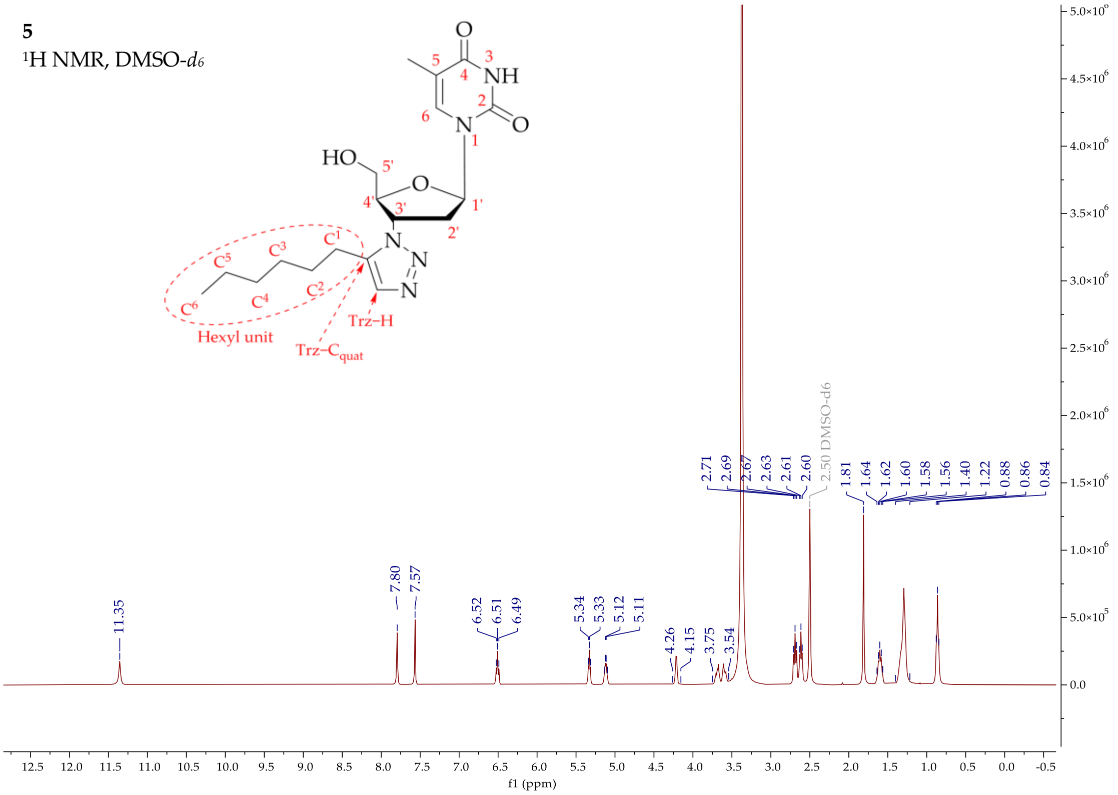

Figure S5. ¹H NMR spectrum of compound **5** recorded in DMSO-d₆.

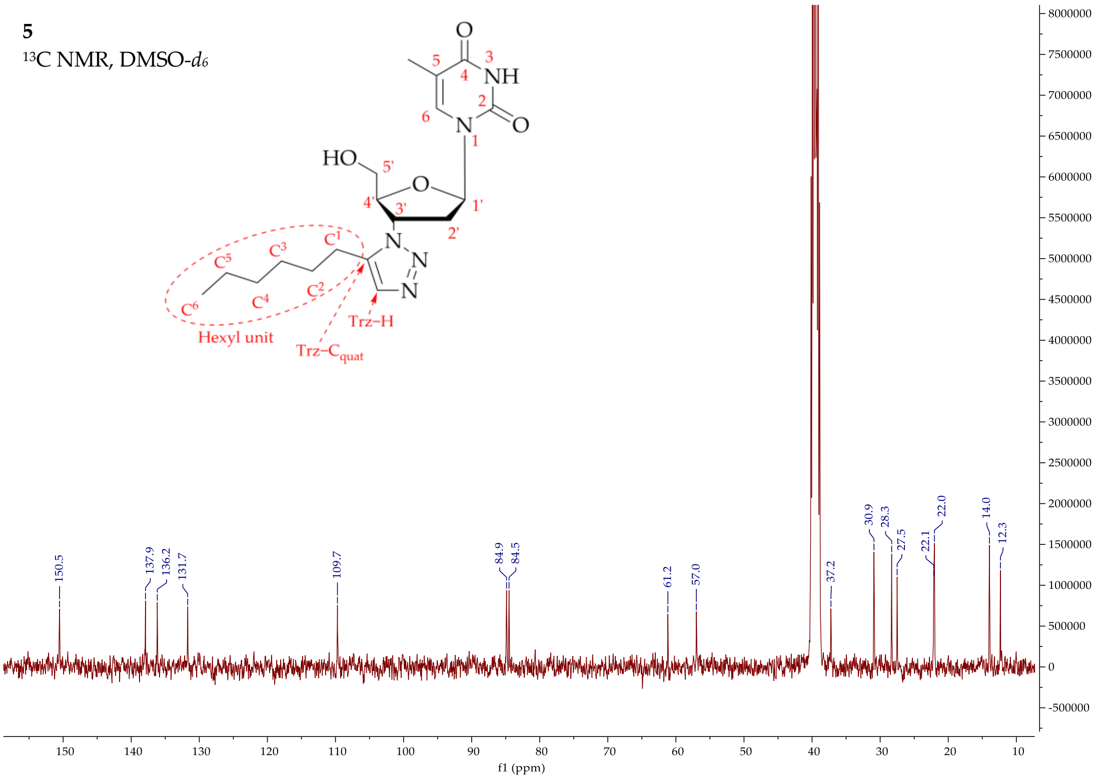

Figure S6. ^{13}C NMR spectrum of compound **5** recorded in DMSO-d_6.

Compound 7

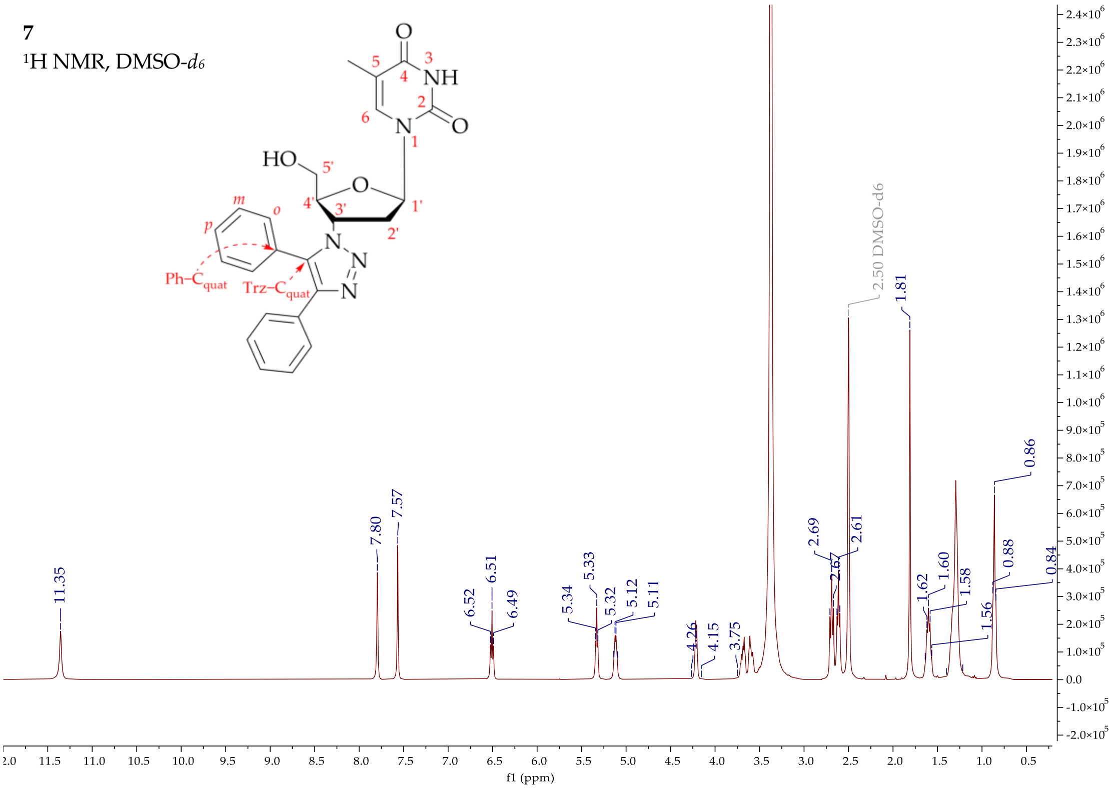

Figure S7. ^{1}H NMR spectrum of compound **7** recorded in DMSO-*d*6.

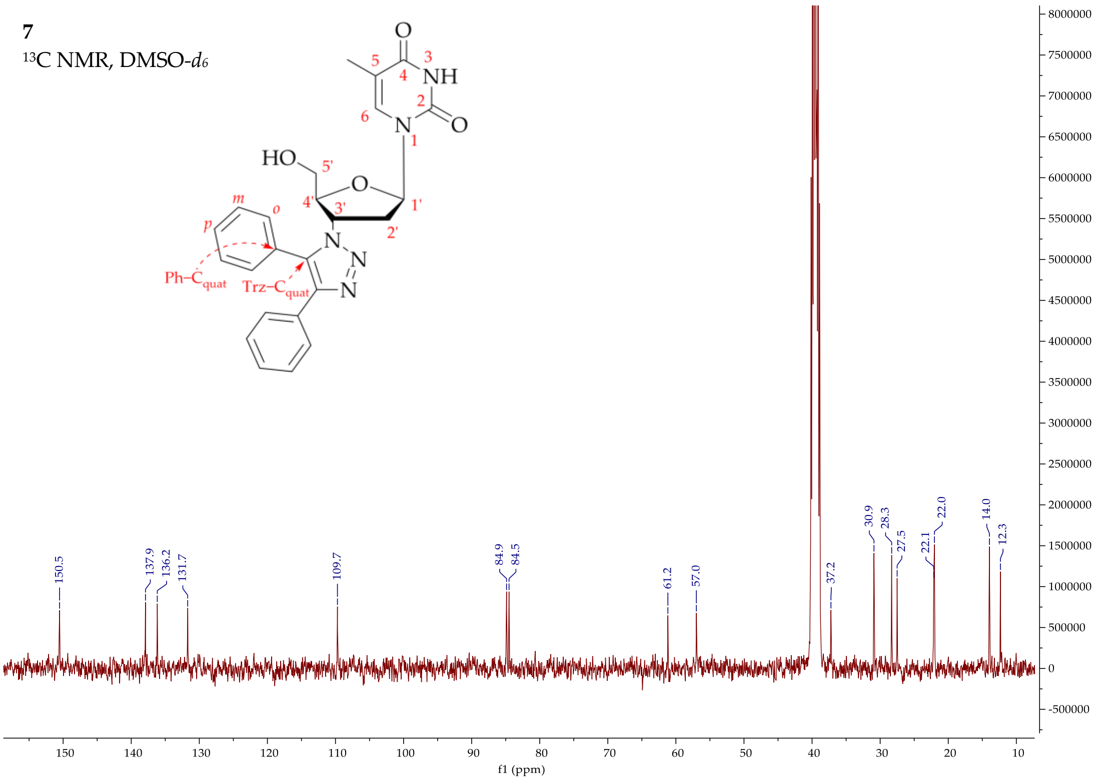

Figure S8. ^{13}C NMR spectrum of compound **7** recorded in DMSO-*d*6.

Compound 8

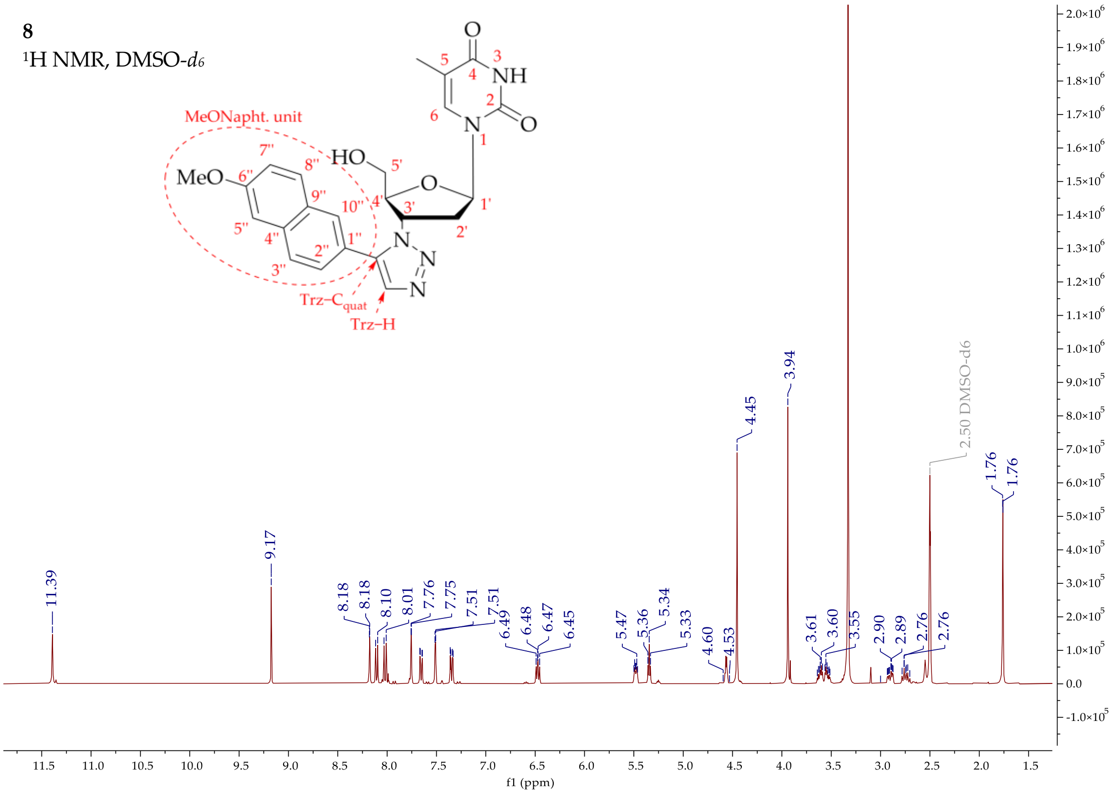

Figure S9. ^{1}H NMR spectrum of compound **8** recorded in DMSO-d_6.

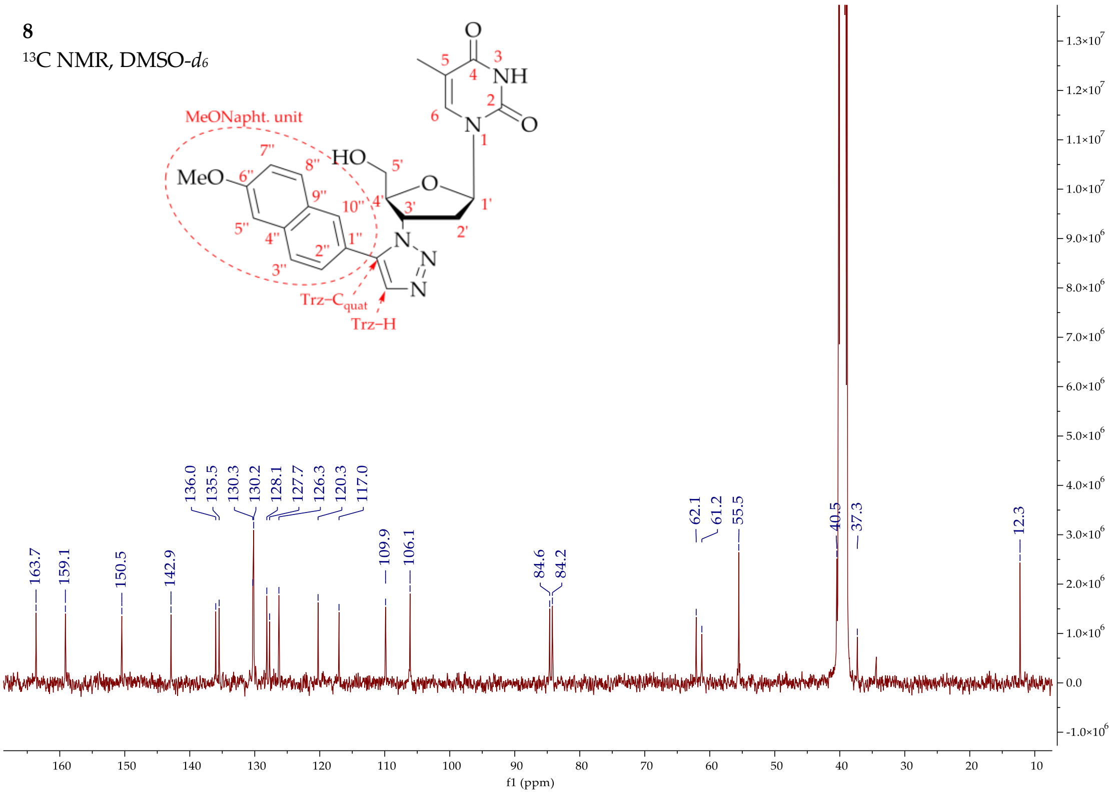

Figure S10. ^{13}C NMR spectrum of compound **8** recorded in DMSO-d_6.

Compound 9

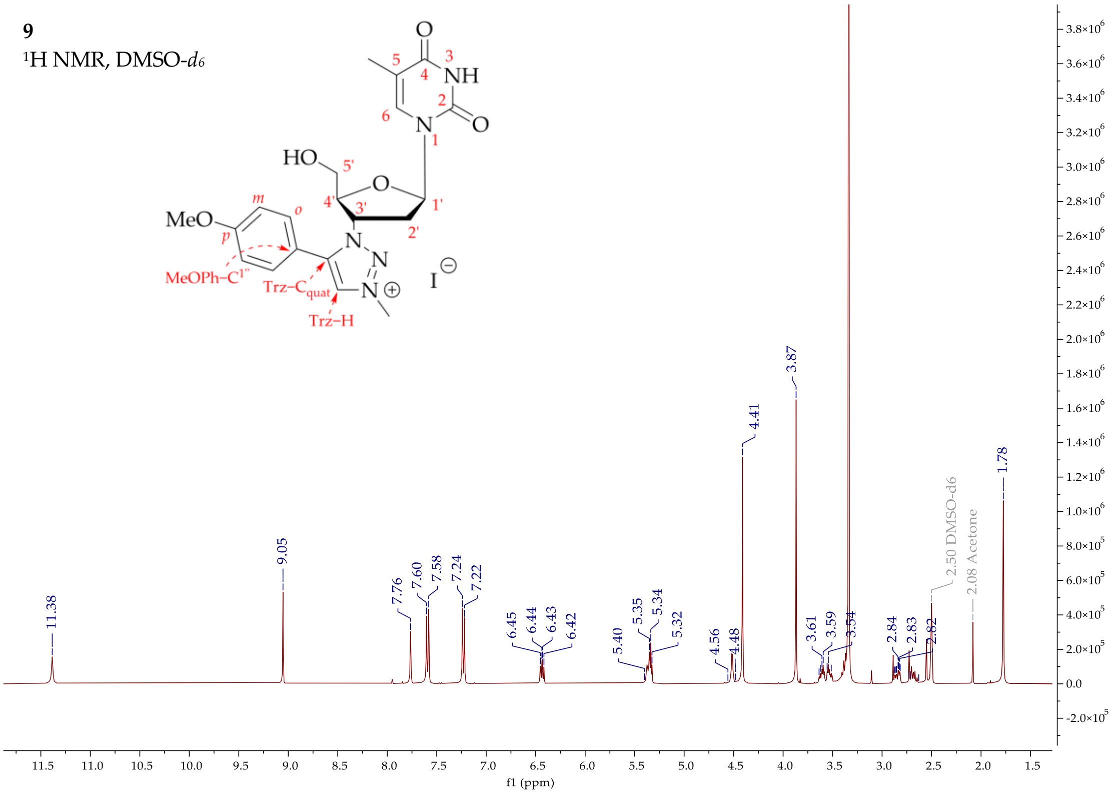

Figure S11. ¹H NMR spectrum of compound **9** recorded in DMSO-*d*₆.

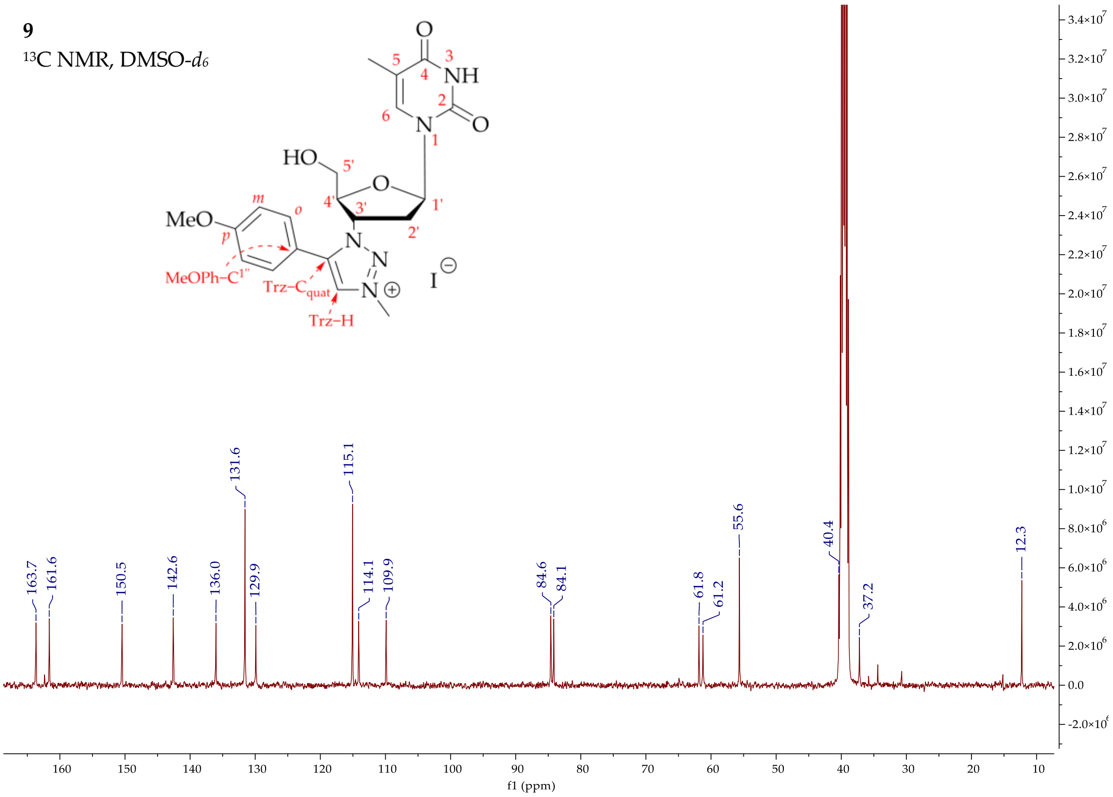

Figure S12. ¹³C NMR spectrum of compound **9** recorded in DMSO-*d*₆.

Compound 10

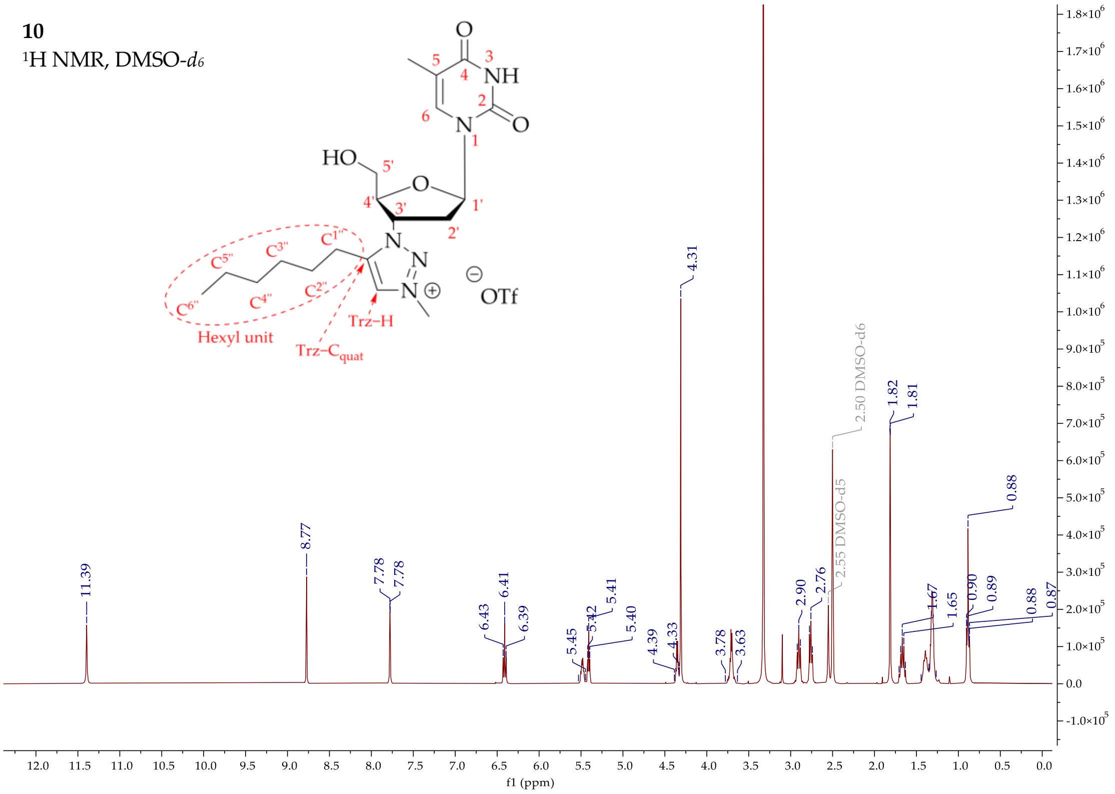

Figure S13. 1H NMR spectrum of compound **10** recorded in DMSO-d6.

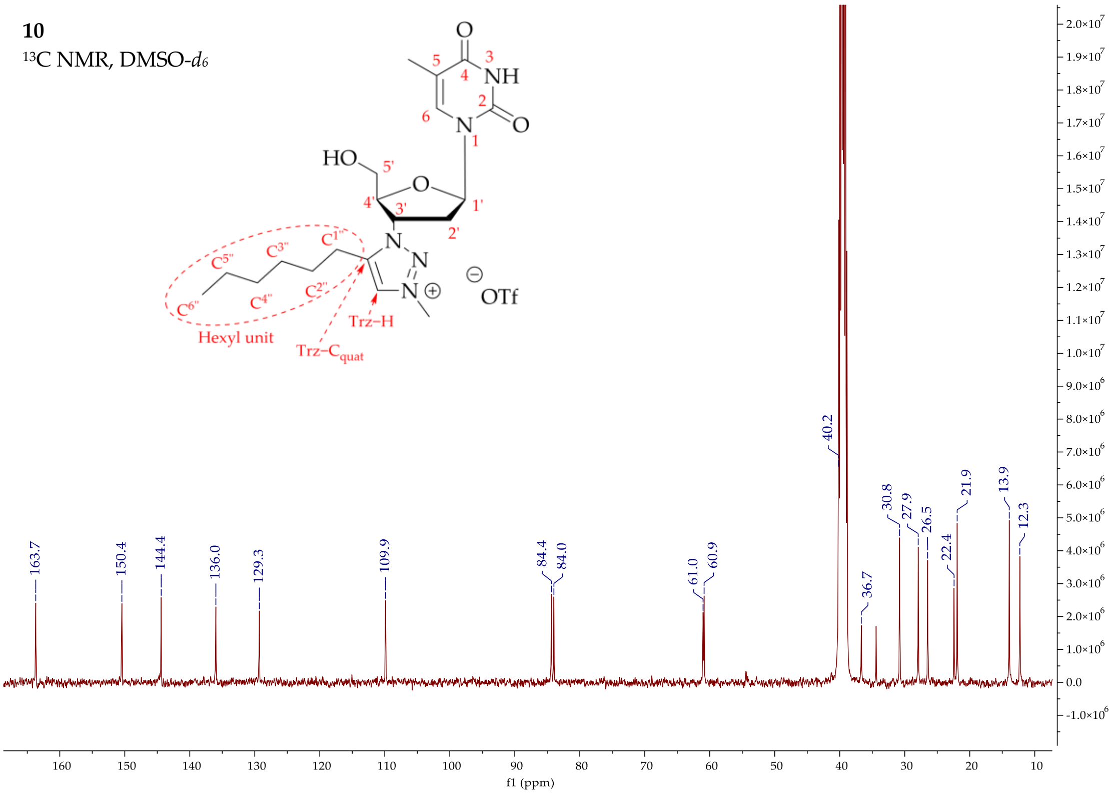

Figure S14. 13C NMR spectrum of compound **10** recorded in DMSO-*d6*.

Compound 11

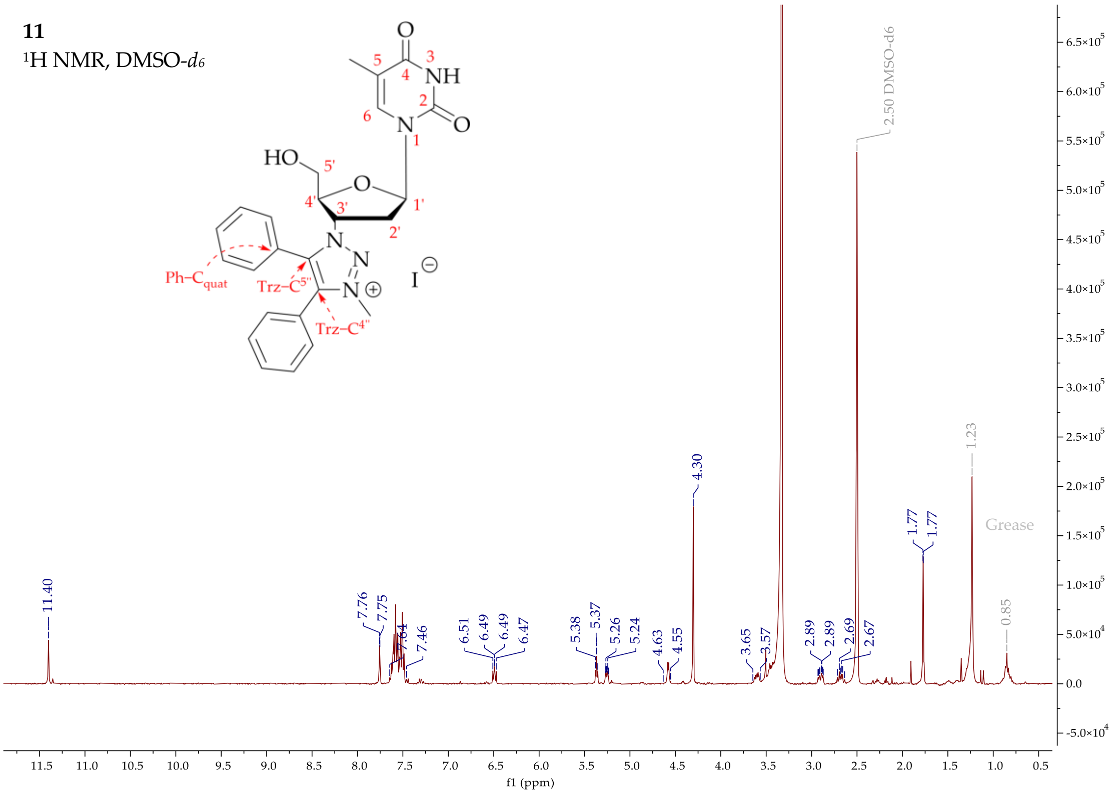

Figure S15. ^{1}H NMR spectrum of compound **11** recorded in DMSO-*d*6.

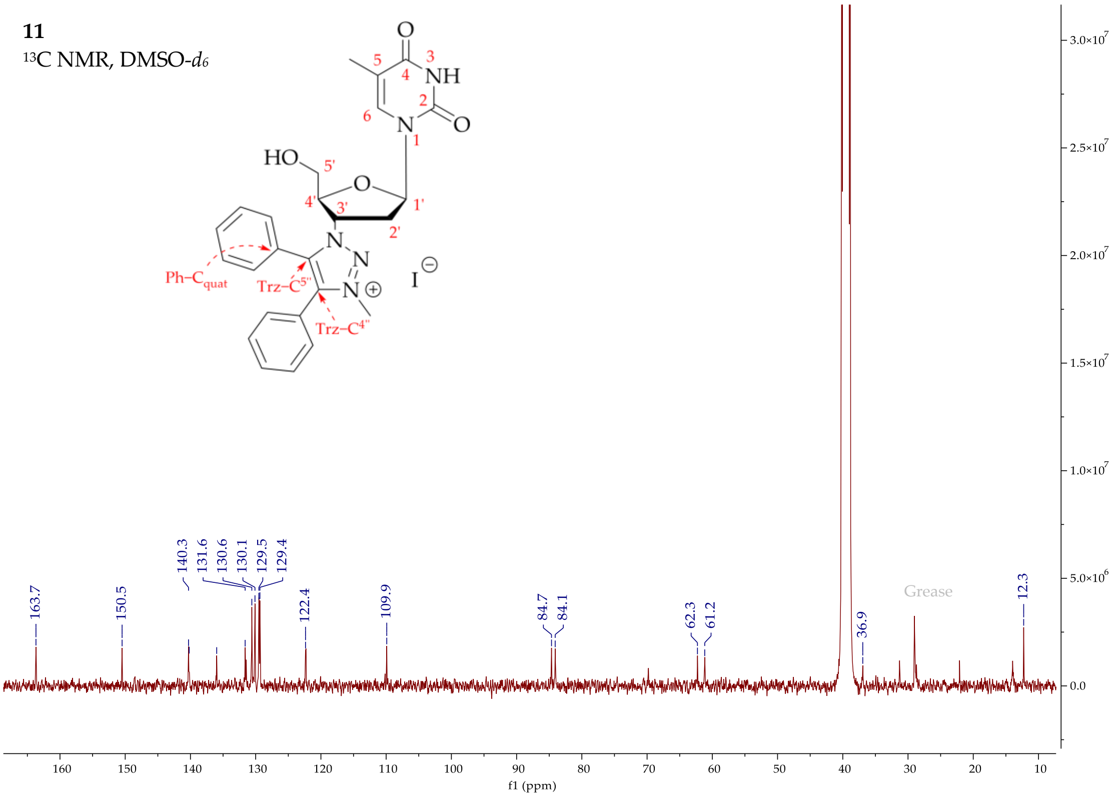

Figure S16. ^{13}C NMR spectrum of compound **11** recorded in DMSO-*d*6.
